# Signatures of anthocyanin metabolites identified in humans inhibit biomarkers of vascular inflammation in human endothelial cells

**DOI:** 10.1002/mnfr.201700053

**Published:** 2017-06-09

**Authors:** Emily F. Warner, Michael J. Smith, Qingzhi Zhang, K. Saki Raheem, David O'Hagan, Maria A. O'Connell, Colin D. Kay

**Affiliations:** ^1^ Department of Nutrition, Norwich Medical School, Bob Champion Research and Education Building University of East Anglia Norwich UK; ^2^ School of Chemistry University of St. Andrews Fife Scotland UK; ^3^ School of Pharmacy University of East Anglia Norwich UK; ^4^ Food Bioprocessing & Nutrition Sciences, Plants for Human Health Institute North Carolina State University Kannapolis NC USA; ^5^ Department of Life Sciences, Faculty of Science and Technology University of Westminster London UK; ^6^ School of Biological Sciences University of East Anglia Norwich UK

**Keywords:** Adhesion, Anthocyanin, Inflammation, Metabolism

## Abstract

**Scope:**

The physiological relevance of contemporary cell culture studies is often perplexing, given the use of unmetabolized phytochemicals at supraphysiological concentrations. We investigated the activity of physiologically relevant anthocyanin metabolite signatures, derived from a previous pharmacokinetics study of 500 mg ^13^C_5_‐cyanidin‐3‐glucoside in eight healthy participants, on soluble vascular adhesion molecule‐1 (VCAM‐1) and interleukin‐6 (IL‐6) in human endothelial cells.

**Methods and results:**

Signatures of peak metabolites (previously identified at 1, 6, and 24 h post‐bolus) were reproduced using pure standards and effects were investigated across concentrations ten‐fold lower and higher than observed mean (<5 μM) serum levels. Tumor necrosis factor‐α (TNF‐α)‐stimulated VCAM‐1 was reduced in response to all treatments, with maximal effects observed for the 6 and 24 h profiles. Profiles tested at ten‐fold below mean serum concentrations (0.19–0.44 μM) remained active. IL‐6 was reduced in response to 1, 6, and 24 h profiles, with maximal effects observed for 6 h and 24 h profiles at concentrations above 2 μM. Protein responses were reflected by reductions in *VCAM‐1* and *IL‐6* mRNA, however there was no effect on phosphorylated NFκB‐p65 expression.

**Conclusion:**

Signatures of anthocyanin metabolites following dietary consumption reduce VCAM‐1 and IL‐6 production, providing evidence of physiologically relevant biological activity.

**Abbreviations**
C3G: cyanidin‐3‐glucoside
HCAEC: human coronary artery endothelial cells
HUVEC: human umbilical vein endothelial cells
IL‐6: interleukin‐6
TNF‐α: tumor necrosis factor‐α
VCAM‐1: vascular adhesion molecule‐1

## 1 Introduction

The consumption of anthocyanins has been linked to a reduced risk of cardiovascular disease 1, 2, though their mechanisms of action are not fully understood. Traditionally, in vitro studies have explored the activity of parent anthocyanins mechanistically, however their low plasma concentrations and rapid clearance kinetics suggests they are not the bioactive forms responsible for in vivo activity. It is therefore probable that anthocyanin bioactivity in vivo results from the lesser studied, though more bioavailable, phenolic metabolites, and we have recently demonstrated they are more active on inflammatory biomarkers than their precursor structures 3, 4, 5.

The understanding of anthocyanin metabolism is relatively contemporary, though it is commonly accepted that their degradation is a result of their chemical instability and the impact of bacterial catabolism, resulting in a number of circulating phenolic metabolites 6, 7. As anthocyanin metabolites do not circulate in isolation following ingestion, but exist as complex mixtures or profiles of metabolites at various concentrations 8, 9, 10, it is important that this is also reflected in the design of experiments exploring the bioactivity of anthocyanins.

**Figure 1:**
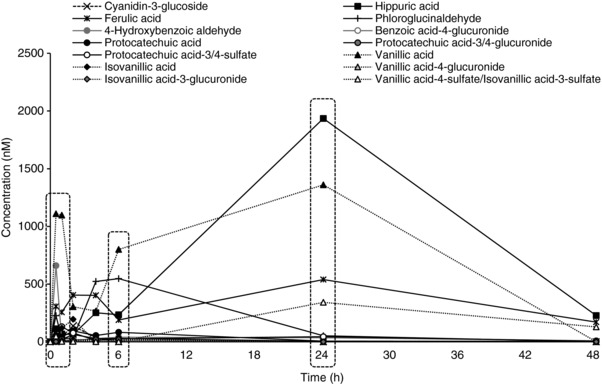
Serum pharmacokinetic signatures of C3G and its metabolites in humans after the consumption of 500 mg ^13^C_5_‐C3G in eight healthy male participants. Data represent mean concentration of specified metabolites from eight participants. Peak signatures (at 1, 6, and 24 h) are indicated by the dashed‐line boxes. “/” indicates isomers quantified together. Common name (chemical name): 4‐hydroxybenzyldehyde (4‐hydroxybenzoicaldehyde); Benzoic acid‐4‐glucuronide (benzoic acid‐4‐O‐glucuronide); Cyanidin‐3‐glucoside (2‐(3,4‐dihydroxyphenyl)‐5,7‐dihydroxy‐3‐chromeniumyl‐β‐D‐glucopyranoside); Ferulic acid (4‐hydroxy‐3‐methoxycinnamic acid); Hippuric acid (N‐benzoylglycine); Isovanillic acid (3‐hydroxy‐4‐methoxybenzoic acid); Isovanillic acid‐3‐glucuronide (4‐methoxybenzoic acid‐3‐O‐glucuronide); Isovanillic acid‐3‐sulfate (4‐methoxybenzoic acid‐3‐sulfate); Phloroglucinaldehyde (2,4,6‐trihydroxybenzaldehyde); Protocatechuic acid (3,4‐dihydroxybenzoic acid); Protocatechuic acid‐3‐sulfate (4‐hydroxybenzoic acid‐3‐sulfate); Protocatechuic acid‐4‐glucuronide (3‐hydroxybenzoic acid‐4‐O‐glucuronide); Protocatechuic acid‐4‐sulfate (3‐hydroxybenzoic acid‐4‐sulfate); Vanillic acid (3‐methoxy‐4‐hydroxybenzoic acid); Vanillic acid‐4‐glucuronide (3‐methoxybenzoic acid‐4‐O‐glucuronide); Vanillic acid‐4‐sulfate (3‐methoxybenzoic acid‐4‐sulfate). Adapted from de Ferrars et al. 11.

The metabolism of a common dietary anthocyanin, cyanidin‐3‐glucoside (C3G), was recently investigated and 29 metabolites were identified following the consumption of 500 mg ^13^C‐labelled cyanidin‐3‐glucoside (C3G) 8, 11. Briefly, eight healthy male participants were fed a single 500 mg oral bolus dose of isotopically labeled C3G (^13^C_5_‐C3G) following a 7‐day washout period (avoiding anthocyanin‐rich foods), where blood was collected at baseline, 0.5, 1, 2, 4, 6, 24, and 48 h. Three distinct peak serum metabolite profiles (or signatures) were observed post consumption at 1, 6, and 24 h (Fig. 1). Similar groupings of metabolites sharing C_max_ and producing distinct biosignatures or peaks of metabolites during clearance have also been observed following consumption of cocoa flavan‐3‐ols 12 and citrus flavanones 13, suggesting this is a common response in the clearance kinetics of flavonoid metabolites. Given that these phenolics circulate at higher concentrations and for longer duration relative to their precursor structures, there is scope to investigate the collective activity of blood profiles of phenolic metabolites on inflammatory processes.

Anthocyanin metabolites have been shown to inhibit the expression of a number of inflammatory biomarkers, such as those involved in vascular adhesion and chemotaxis, including soluble vascular cellular adhesion molecule‐1 (VCAM‐1) and interleukin‐6 (IL‐6) 14, 15, both of which are markers of cardiovascular disease risk and mortality 16, 17 and logical targets for exploring the potential mechanisms of action of anthocyanin metabolites. The aims of the present study were therefore: investigate the effects of unique C3G metabolite signatures, observed to peak in vivo at 1, 6, and 24 h post consumption, on VCAM‐1 and IL‐6 protein secretion by two cell types, human umbilical vein endothelial cells (HUVEC) and human coronary artery endothelial cells (HCAECs); investigate the effects of metabolite signatures across a range of concentrations, reflecting levels ten‐fold lower (<0.5 μM) and ten‐fold higher (<50 μM) than mean concentrations (<5 μM) observed previously 8, 11; finally, identify mechanistic effects on VCAM*‐1* and IL‐6 mRNA by targeting a key inflammatory transcriptional target, NFκB.

## 2 Methods

### 2.1 Materials

Early passage human umbilical vein endothelial cells (HUVECs) (cryopreserved, pooled donors, passage 2), large vessel endothelial growth medium (containing 2% fetal calf serum, human epidermal growth factor, human fibroblast growth factor, 25 μg/mL gentamycin, 50  ng/mL amphotericin, hydrocortisone and heparin) and trypsin passage pack were purchased from Caltag Medsystems (Buckingham, UK). Early passage (passage 2) human coronary artery endothelial cells (HCAECs) (cryopreserved, single donors), endothelial cell medium MV (containing 5% fetal calf serum, endothelial cell growth supplement, recombinant human epidermal growth factor, heparin, and hydrocortisone) and Detach Kit were purchased from PromoCell GmbH (Heidelberg, Germany). The conjugated metabolites, as listed in Table 1, were synthesized at the University of St. Andrews (UK) 18. Human‐derived fibronectin, TNF‐α, and all flavonoids and unconjugated phenolic acids (Table 1) were obtained from Sigma Aldrich (Dorset, UK), with the exception of cyanidin‐3‐glucoside (Extrasynthase, France).

**Table 1:** Serum profile constituents and concentrations

Analyte	Final profile concentration (nM)								
	1 h profile			6 h profile			24 h profile		
4‐Hydroxybenzyldehyde	1	10	100	1	10	100	1	10	100
Benzoic acid‐4‐glucuronide	1	10	100	4	40	400	4	40	400
Cyanidin‐3‐glucoside	5	50	500	0	0	0	0	0	0
Ferulic acid	29	290	2900	21	210	2100	59	590	5900
Hippuric acid	7	70	700	23	230	2300	194	1940	19 400
Isovanillic acid	12	120	1200	0	0	0	0	0	0
Isovanillic acid‐3‐glucuronide	1	10	100	2	20	200	0	0	0
Isovanillic acid‐3‐sulfate	0	0	0	0	0	0	17	170	1700
Phloroglucinaldehyde	3	30	300	55	550	5500	5	50	500
Protocatechuic acid	4	40	400	8	80	800	1	10	100
Protocatechuic acid‐3‐sulfate	7	70	700	2	20	200	2	20	200
Protocatechuic acid‐4‐glucuronide	2	20	200	3	30	300	0	0	0
Protocatechuic acid‐4‐sulfate	7	70	700	2	20	200	2	20	200
Vanillic acid	110	1100	11 000	80	800	8000	136	1360	13 600
Vanillic acid‐O‐4‐glucuronide	1	10	100	2	20	200	0	0	0
Vanillic acid‐4‐sulfate	0	0	0	0	0	0	17	170	1700
**Total**	**190**	**1900**	**19 000**	**203**	**2030**	**20 300**	**438**	**4380**	**43 800**

Common name (chemical name): 4‐hydroxybenzyldehyde (4‐hydroxybenzoicaldehyde); Benzoic acid‐4‐glucuronide (benzoic acid‐4‐O‐glucuronide); Cyanidin‐3‐glucoside (2‐(3,4‐dihydroxyphenyl)‐5,7‐dihydroxy‐3‐chromeniumyl‐β‐D‐glucopyranoside); Ferulic acid (4‐hydroxy‐3‐methoxycinnamic acid); Hippuric acid (N‐benzoylglycine); Isovanillic acid (3‐hydroxy‐4‐methoxybenzoic acid); Isovanillic acid‐3‐glucuronide (4‐methoxybenzoic acid‐3‐O‐glucuronide); Isovanillic acid‐3‐sulfate (4‐methoxybenzoic acid‐3‐sulfate); Phloroglucinaldehyde (2,4,6‐trihydroxybenzaldehyde); Protocatechuic acid (3,4‐dihydroxybenzoic acid); Protocatechuic acid‐3‐sulfate (4‐hydroxybenzoic acid‐3‐sulfate); Protocatechuic acid‐4‐glucuronide (3‐hydroxybenzoic acid‐4‐O‐glucuronide); Protocatechuic acid‐4‐sulfate (3‐hydroxybenzoic acid‐4‐sulfate); Vanillic acid (3‐methoxy‐4‐hydroxybenzoic acid); Vanillic acid‐4‐glucuronide (3‐methoxybenzoic acid‐4‐O‐glucuronide); Vanillic acid‐4‐sulfate (3‐methoxybenzoic acid‐4‐sulfate).

### 2.2 Treatment metabolite profiles

Stock solutions for cell culture experiments were prepared in 100% DMSO at 200 mM and stored at ‐80°C with the exception of cyanidin‐3‐glucoside, which was prepared at 40 mM, and the sulfate‐ conjugated phenolic acids, which were prepared at 25 mM in 50% DMSO (50% PBS) to maintain stability whilst reducing final DMSO concentrations in working solutions. Working solutions of 1 mM were prepared in supplemented media before being diluted to their working concentrations (Table 1) and stored at 4°C until experimental commencement (with the exception of cyanidin‐3‐glucoside, which was added immediately prior to the final dilutions to maintain stability). Solutions were subsequently diluted in supplemented media as required (Table 1) immediately prior to experiment commencement.

### 2.3 Cell culture

HUVECs and HCAECs were routinely cultured in fibronectin coated T75 flasks (0.25 μg/cm^2^), using large vessel endothelial growth medium and endothelial cell medium MV, respectively, at 37°C and 5% CO_2_. Cells were sub‐cultured using a trypsin passage pack or Detach Kit, according to the manufacturer's instructions. HUVECs were used at passage 4 and HCAECs were used between passages 3 and 6. All cells were incubated in supplemented media for 24 h at 37°C, 5% CO_2_, in a humidified atmosphere, prior to experiment commencement.

### 2.4 VCAM‐1 and IL‐6 protein expression

HUVEC or HCAEC were seeded at 80 000 cells/well in fibronectin coated 24‐well plates. Cells were treated for 30 min with peak metabolite profiles identified previously at 1, 6, 24 h post consumption (Table 1) or 0.01% DMSO (vehicle control) prior to the addition of 10 ng/mL (HUVEC) or 0.1 ng/mL (HCAEC) TNF‐α (determined as providing maximal induction while maintaining physiologically relevant concentrations following time‐ and concentration‐response experiments), and incubated for 24 h at 37°C, 5% CO_2_, in a humidified atmosphere. Supernatants were collected on ice, centrifuged at 2000 ×*g* for 10 min at 4°C, and stored at ‐80°C prior to ELISA. Samples were thawed at room temperature and vortexed for 3 × 5 s immediately prior to analysis. Supernatants were diluted 1:1 in Reagent Diluent (R&D Systems) and protein expression of soluble VCAM‐1 (sVCAM‐1; hereby referred to as VCAM‐1) and IL‐6 were determined by commercially available DuoSet enzyme‐linked immunosorbent assay (ELISA) (R&D Systems), according to the manufacturer's instructions. Absorbance values for all ELISA plates were recorded using an OMEGA plate reader from BMG LABTECH (Bucks, UK).

### 2.5 *VCAM‐1* and *IL‐6* mRNA expression

HCAEC were seeded at 200 000 cells/well in fibronectin coated 6‐well plates. Cells were pre‐treated for 30 min with the highest working concentrations of the metabolite profiles (19, 20, 44 μM, reflecting serum concentrations at 1, 6, 24 h respectively) of each serum profile or 0.01% DMSO (vehicle control) prior to the addition of 0.1  ng/mL TNF‐α, and incubated for 4 h at 37°C, 5% CO_2_, in a humidified atmosphere. Cell culture supernatants were removed and cells washed 3× with PBS. Total RNA was extracted from HUVECs and reverse transcribed to cDNA using conditions previously described 3. Real‐time quantitative PCR (RT‐qPCR) was carried out using 25 ng cDNA with the addition of *VCAM‐1* primers (forward primer, 5’‐CAGGCTAAGTTACATATTGATGACAT‐3’; reverse primer, 5’‐GAGGAAGGGCTGACCAAGAC‐3’), or *IL‐6* primers (forward primer, 5’‐GCAGAAAACAACCTGAACCTT; reverse primer, 3’‐ACCTCAAACTCCAAAAGACCA‐5’) and real‐time PCR Precision master mix with SYBR green (Primer Design, UK). RT‐qPCR was carried out using the ABI7500 (Applied Biosystems, UK) system, using cycle methods previously described 3. Relative changes in gene expression from the TNF‐α control were quantified using the comparative Ct method 19. The difference between recorded Ct values for treatment and positive control samples were calculated in the first instance for all genes. geNORM analysis was carried out using qbasePLUS software (version 2.3; Biogazelle, Belgium) to determine stable reference genes (*VIPAS39* and *PRDM4*), the geometric mean of which were used to normalize the data in subsequent experiments.

### 2.6 Phospho‐NFκB p65 expression

HCAEC were seeded at 200 000 cells/well in fibronectin coated 6‐well plates. Cells were pre‐treated for 30 min with the highest working concentrations of each serum profile (19, 20, 44 μM, reflecting 1, 6, and 24 h serum concentrations respectively) or 0.01% DMSO (vehicle control) prior to the addition of 10  ng/mL TNF‐α, and incubated for 15 min at 37°C, 5% CO_2_, in a humidified atmosphere. Cells were washed 3× with PBS and lysed with NP‐40 lysis buffer; total protein concentrations were determined by BCA assay, and proteins were separated and probed by SDS‐PAGE and Western blotting, respectively, as described previously by this group 20. Primary antibody solution contained 0.1% PBS with 20% T20 Blocking Buffer (Thermoscientific, UK), with rabbit polyclonal anti‐NFκB p65 (Ser536) antibody (ab28856; Abcam, UK; 1:2000 dilution) and chicken polyclonal anti‐GAPDH (AB2302; Millipore, UK; 1:15 000 dilution) and secondary antibody solutions contained 0.1% PBS with 20% T20 Blocking Buffer and 0.1% SDS, with goat anti‐rabbit (IRdye 800CW; Li‐Cor, UK; 1:15 000 dilution) and donkey‐anti‐chicken (IRdye 680LT; Li‐Cor, UK; 1:15 000 dilution). Membranes were imaged and quantified by densitometry at 700 nm and 800 nm using Odyssey Infrared Imaging System and Odyssey Infrared Imaging System Application Software, respectively (Li‐Cor; version 3.0.21).

### 2.7 Data analysis

VCAM‐1 and IL‐6 protein (pg/mL) or mRNA (fold change) were recorded as the mean of two technical duplicates, and reported relative to the TNF‐α positive control (containing TNF‐α without DMSO) as the mean of three independent experiments. Phospho‐NFκB p65 expression (infrared density) data were normalized to GAPDH reference gene and data were presented graphically as a fold change of vehicle control (DMSO). Treatment effects were established by one‐way analysis of variance (ANOVA) with post‐hoc least significant difference (LSD) conducted using SPSS for Windows (version 22.0; IBM, New York, USA). Untreated controls were not included in the ANOVA for treatment effect but presented graphically, where Students *t*‐test established difference relative to vehicle control (DMSO). All data represent the mean ± SD of three biological replicates (*n* = 3).

## 3 Results

### 3.1 Effect of peak cyanidin‐3‐glucoside metabolite signatures on VCAM‐1 protein expression

**Figure 2:**
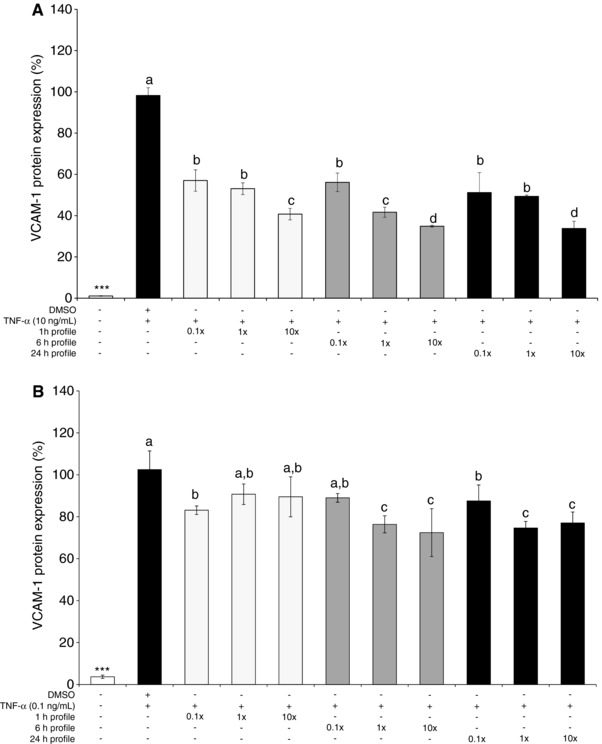
Effect of peak metabolite signatures on TNF‐α stimulated VCAM‐1 secretion by A) HUVEC, B) HCAEC. Cells were treated with three concentrations of three serum metabolite profiles (representing ten‐fold lower and ten‐fold higher concentrations than the mean concentrations observed by Czank et al. 8; Table 1) prior to the addition of 10 or 0.1  ng/mL TNF‐α for 24 h. Data were normalized to a TNF‐α control (no DMSO) and columns represent the mean ± SD, *n* = 3 biological replicates. Labeled means without a common letter differ, *p* ≤ 0.05 (ANOVA with post hoc LSD). *Different from DMSO, p ≤ 0.05 (t‐test). Abbreviations: HUVEC, human umbilical vein endothelial cells; TNF‐α, tumor necrosis factor‐α; VCAM‐1, soluble vascular adhesion molecule‐1.

The effects of signatures of cyanidin‐3‐glucoside metabolites (Fig. 1) on TNF‐α stimulated VCAM‐1 secretion was explored at the mean concentrations observed clinically, as well as concentrations ten‐fold lower and ten‐fold higher (Table 1), in HUVEC and validated in HCAEC. No treatments were cytotoxic at any tested concentration as established utilizing the WST‐1 cytotoxicity assay (Roche, United Kingdom). HUVEC‐secreted VCAM‐1 (Fig. 2A) was reduced relative to the vehicle control in response to all treatments at all tested concentrations, with the maximal effects observed for 6 and 24 h metabolite signatures (−65.12 ± 0.37% and −66.24 ± 2.88, respectively; *p* ≤ 0.001) at cumulative concentrations of 20 and 44 μM (respectively). The activity expressed in HCAEC was slightly less than that of the HUVEC, however similarly, inhibition of HCAEC‐secreted VCAM‐1 (Fig. 2B) was greatest for treatments reflecting the 6 and 24 h metabolite signatures (−30.07 ± 11.41% and −27.84 ± 3.09%, respectively; *p* ≤ 0.001). For both cell lines, even the profiles tested at concentrations 10‐fold below the mean serum levels reported (0.19, 0.20, 0.44 μM reflecting serum profiles at 1, 6, and 24 h, respectively) significantly reduced VCAM‐1 secretion (*p* ≤ 0.05).

### 3.2 Effect of peak cyanidin‐3‐glucoside metabolite signatures on IL‐6 protein expression

**Figure 3:**
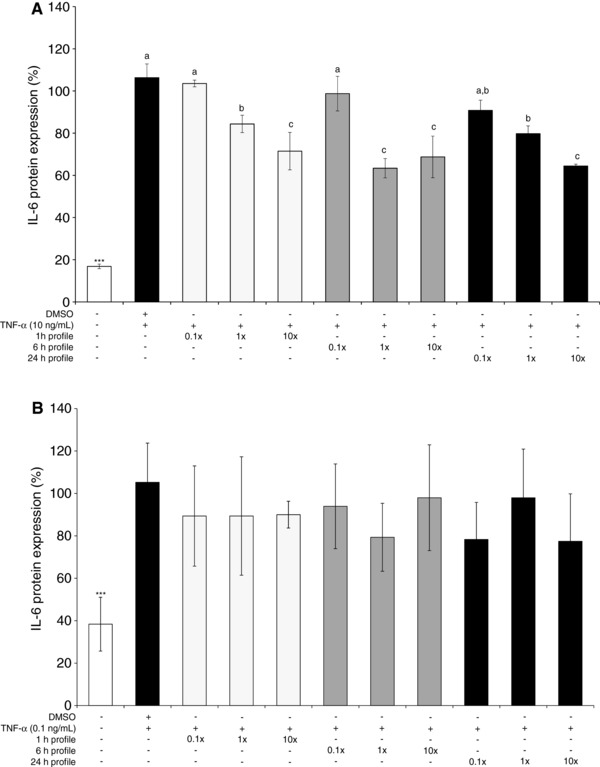
Effect of peak metabolite signatures on TNF‐α stimulated IL‐6 secretion by A) HUVEC, B) HCAEC. Cells were treated with three concentrations of three serum metabolite profiles (representing ten‐fold lower and ten‐fold higher concentrations than the mean concentrations observed by Czank et al. 8; Table 1) prior to the addition of 10 or 0.1  ng/mL TNF‐α for 24 h. Data were normalized to a TNF‐α control (no DMSO) and columns represent the mean ± SD, *n* = 3 biological replicates. Labeled means without a common letter differ, *p* ≤ 0.05 (ANOVA with post hoc LSD). *Different from DMSO, *p* ≤ 0.05 (*t*‐test). Abbreviations: HCAEC, human coronary artery endothelial cells; TNF‐α, tumor necrosis factor‐α; IL‐6, interleukin‐6.

The effects of cyanidin‐3‐glucoside metabolite signatures (Fig. 1) on TNF‐α stimulated IL‐6 secretion was explored at the mean concentration observed clinically (Table 1), as well as ten‐fold lower and ten‐fold higher concentrations in HUVEC and validated in HCAEC. HUVEC‐secreted IL‐6 (Fig. 3A) was reduced relative to the vehicle control in response to all treatments, with the exception of the concentrations tested 10‐fold below the mean serum levels (0.19, 0.20, 0.44 μM reflecting serum concentrations at 1, 6, and 24 h, respectively). Maximal effects on IL‐6 were observed in response to the three metabolite signatures (1, 6, and 24 h) at cumulative concentrations of 2, 20, and 44 μM, respectively (−36.63 ± 3.73%, −31.26 ± 8.06%, −35.56 ± 0.70%; *p* ≤ 0.001). Activity was not reduced in HCAEC in response to any treatment (*p* > 0.05; Fig. 3B). Maximal concentrations of each metabolite signature (19, 20, 44 μM), reflecting serum concentrations at 1, 6, and 24 h (respectively) were taken forward to confirm their effect on VCAM‐1 and IL‐6 mRNA in HCAEC.

### 3.3 Effect of peak cyanidin‐3‐glucoside metabolite profiles on *VCAM‐1* and *IL‐6* mRNA expression

**Figure 4:**
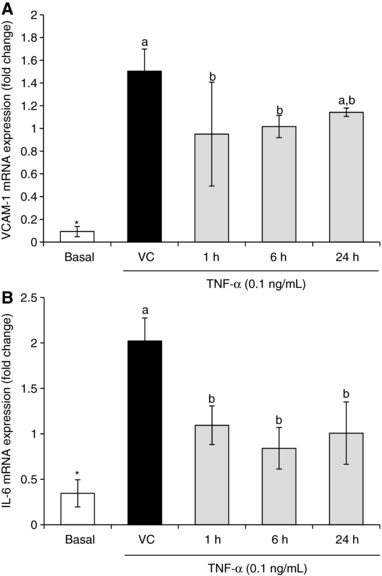
Effect of peak metabolite signatures on TNF‐α stimulated *VCAM‐1* and *IL‐6* mRNA expression in HCAEC (A) *VCAM‐1*, (B) *IL‐6*. Cells were treated with the highest concentration signature metabolites (19, 20, 44 μM reflecting 1, 6, and 24 h serum profiles respectively; Czank et al. 8) and stimulated with 0.1  ng/mL TNF‐α for 4 h. Amplification values were normalized to the geometric mean of two stable reference genes, *VIPAS39* and *PRDM4*. Data were normalized to a TNF‐α control (no DMSO) and columns represent the mean ± SD, *n* = 3 biological replicates. Labeled means without a common letter differ significantly, *p* ≤ 0.05 (ANOVA with post hoc LSD). *Different from DMSO, *p* ≤ 0.05 (*t*‐test). Abbreviations: HCAEC, human coronary artery endothelial cells; TNF‐α, tumor necrosis factor‐α; IL‐6, interleukin‐6; VCAM‐1, vascular adhesion molecule‐1.

Peak metabolite signatures were used to determine whether TNF‐α stimulated *VCAM‐1* and *IL‐6* protein secretion was reflected by mRNA expression in HCAEC (Fig. 4). Here TNF‐α stimulated *VCAM‐1* mRNA expression was reduced by 0.55 ± 0.25 fold, 0.49 ± 0.13 fold, and 0.36 ± 0.21 fold in response to 19, 20, 44 μM concentrations as observed clinically at 1, 6, and 24 h (respectively), compared to the vehicle control (Fig. 4A). TNF‐α stimulated *IL‐6* mRNA expression was reduced by 0.93 ± 0.10 fold, 1.18 ± 0.30 fold, and 1.01 ± 0.54 fold in response to 19, 20, and 44 μM treatment profiles as observed clinically at 1, 6, and 24 h, respectively (*p* ≤ 0.05; Fig. 4B).

### 3.4 Effect of peak cyanidin‐3‐glucoside metabolite signatures on phosphorylated NFκB p65 expression

**Figure 5:**
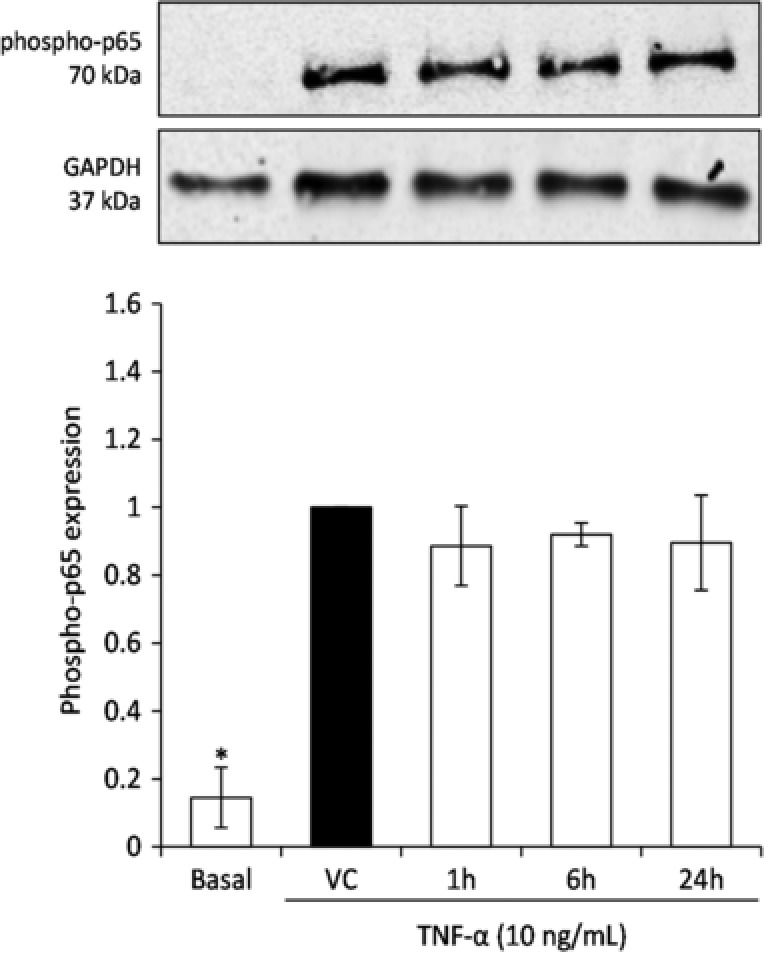
Effect of peak metabolite signatures on TNF‐α stimulated phosphor‐NFκB p65 expression in HCAEC. Cells were treated with the highest concentration signature metabolites (19, 20, 44 reflecting 1, 6, and 24 h serum profiles respectively; Czank et al. 8), and stimulated with 10  ng/mL TNF‐α for 15 min. Data were normalized to the vehicle control (DMSO) and columns represent the mean ± SD, n = 3 biological replicates. Blots are representative of one of three replicates. Density values were normalized to reference protein, GAPDH. Labeled means without a common letter differ significantly, *p* ≤ 0.05 (ANOVA with post hoc LSD). Comparisons of untreated cells to vehicle control (DMSO) were established via Student's *t*‐test, ^*^ *p* ≤ 0.05. Abbreviations: HCAEC, human coronary artery endothelial cells; TNF‐α, tumor necrosis factor‐α.

Peak metabolite signatures were further explored for their effect on TNF‐α stimulated NFκB transcription factor p65 in HCAEC (Fig. 5), where there was no apparent activity beyond that of the vehicle control (*p* > 0.05).

## 4 Discussion

The present study is the first to explore the activity of physiologically relevant signatures of anthocyanin metabolites. Here we utilized three unique treatments based on peak concentrations observed in serum post consumption (Fig. 1) 8, 11, with the aim of elucidating in vivo activity. Peak signatures of metabolites (Table 1) displayed significant inhibitory effects on VCAM‐1 protein secretion (Fig. 2) at concentrations observed in vivo 11, suggesting physiologically achievable bioactivity. The greatest inhibition of VCAM‐1 was observed in response to the 24 h metabolite signature, suggesting metabolites of lower intestinal microbial origin are responsible for fasting or chronic anti‐inflammatory effects.

Peak metabolite signatures appeared to have biological activity, despite the extremely low concentrations of their individual constitutes. Here responses were often greater then activities previously reported by our group for the same metabolites in isolation or in mixtures at equal molar concentrations 4, 5. Previous studies have explored this concept of physiologically relevant concentrations of metabolite profiles by applying extracted serum/plasma to cell culture models. For example, Koga et al. found serum containing metabolites of (+)‐catechin significantly reduced U937 adhesion to human aortic endothelial cells (HAEC) relative to the pure metabolites in isolation 21. Other studies have used both extracted animal and human plasma in a similar manner 22, 23, 24, however the limitation of this study design is that plasma contains many bioactive constituents other than flavonoid metabolites, making it difficult to compare treatments to controls and eliminate confounders stemming from varying endogenous analytes. There is scope to validate the present findings using this model, however, these limitations still exist. The novelty of the present study is that the experiments can be appropriately controlled for vehicle, which allows a more direct exploration of mechanism of action. It could however be argued that this in itself is a limitation, as metabolites may act differentially in serum relative in cell culture media; Indeed, certain flavonoids have been shown to interact with serum albumin in in vitro or ex vivo conditions 25. Furthermore, in utilizing average reported metabolite concentrations, compositions do not reflect individual participants’ blood profiles. Finally, the study design cannot capture the impact of other blood metabolites/analytes escaping sample preparation and detection methodologies.

The highest mean concentration of metabolites detected following the 500 mg bolus of ^13^C‐labeled anthocyanins (equivalent to the consumption of approximately 100 g of blackberries 26) was observed at 24 h post consumption and totaled 4.38 μM 11. In this study, a high inter‐individual variation in metabolism was observed. For example, the serum C_max_ for hippuric acid was 1962 ± 1389 nM, indicating the mean concentration varies greatly between individuals and could be in excess of 3000 nM for a single metabolite. The present study sought to address this issue by utilizing treatment concentrations reflecting the lowest and highest concentrations reported between individuals (0.80–13.18 μM 8, 11). As such, we used three metabolite concentrations, representing 10‐fold higher and lower concentrations then the observed mean. Surprisingly, there was very little difference in the inhibition of VCAM‐1 protein expression between the mean and ten‐fold lower and higher concentrations of the metabolites. This suggests that either there is a threshold activity or that there is something unique about these mixtures of metabolites which, when combined, have some additive or synergistic activity. This outcome is important as these concentrations reflect dietary achievable levels 27. As phenolic metabolites are common to a number of dietary flavonoids and food sources 28, it is possible that metabolite signature concentrations utilized in the present study could be exceeded following a habitual polyphenol‐rich diet, given that consumption of polyphenols in Europe has been estimated between 744–1786 mg/day 29. As effects were observed at the lowest concentrations in the present study (between 0.19 and 0.44 μM), even low levels of dietary polyphenol consumption would have beneficial effects on inflammatory status.

Although peak metabolite profiles inhibited IL‐6 secretion at 1, 6, and 24 h in HUVEC, this was not reflected in HCAEC, though it is possible that effects were masked by the large variation between replicates. It is possible that the reduced stimulus (TNF‐α) concentration (0.1  ng/mL in the present study relative to 10  ng/mL) increased variation as a result of low IL‐6 induction, making it difficult to quantify significant activity.

The effects of the treatments on VCAM‐1 and IL‐6 mRNA expression were investigated to determine whether these would reflect changes in protein expression. In our recent study 5, it was observed that only protocatechuic acid (PCA) inhibited *VCAM‐1* mRNA expression at the highest tested concentration, 100 μM. It is interesting that *VCAM‐1* and *IL‐6* mRNAs were reduced by half in response to the three metabolite profiles which reflect cumulative total metabolite concentrations of only 19, 23, and 44 μM, respectively. Given the low concentrations of metabolites in the present treatment mixtures, it appears that certain metabolites are acting additively or synergistically, potentially through effecting multiple pathways simultaneously. Multiple pathways are indeed thought to be affected following anthocyanin metabolite treatment, for example, aortas of ApoE‐deficient mice fed an anthocyanin‐rich bilberry extract, demonstrated the modulation of 1261 genes which code for proteins involved in the regulation of cellular processes, including adhesion and inflammatory biomarker expression 14.

NFκB is a key transcription factor pathway in the TNF‐α stimulated expression of adhesion molecules in endothelial cells 30. In the present study, no effect was observed on the expression of phosphorylated p65, suggesting alternative mechanisms which influence adhesion molecule expression are at play, such as AP‐1 activity via p38 and JNK MAP kinase. A recent study of ours 5 as well as that of Krga et al. 31 also demonstrated no activity of flavonoid metabolites on NFκB.

Data from the present study suggest that the metabolite profile with the maximum inflammatory effect was observed at 24 h post‐consumption, suggesting fasting or chronic effects are possible. Conversely, improvements in flow‐mediated dilation (FMD) and blood pressure in response to feeding anthocyanins are most often observed acutely, between 1 and 6 h post‐consumption (maximum response at 2 h) 32. In the study of Rodriguez‐Mateos et al. 32, following the consumption of a drink containing blueberry anthocyanins, benzoic and vanillic acids positively correlated with FMD at 1–2 h, whereas hippuric, hydroxyhippuric, and homovanillic acids correlated with the FMD at 6 h. These data suggests an acute‐phase modulatory vascular response of phenol metabolites. Based on these findings and those of the present study it is possible that sudden vascular responses are mediated by very low levels of parent flavonoids and their rapid degradation products, which are succeeded by a delayed anti‐inflammatory response, mediated by products of lower intestinal bacterial catabolism and hepatic phase II conjugation. This ultimately suggests a dual mechanistic activity of flavonoids. This hypothesis requires conformation in randomized‐control trials designed having inflammation as a primary endpoint and utilizing populations both responsive to inflammatory and vascular intervention following dietary manipulation (i.e., neither completely healthy nor chronically unhealthy), as flavonoids are likely to be effective as a preventative strategy rather than a pharmacological therapy.

In conclusion, the present study identified that signatures of anthocyanin metabolites, identified post consumption of dietary achievable levels of anthocyanins, have inhibitory effects on inflammatory protein secretion. Further work is required to elucidate the multiple mechanisms potentially at play, ultimately informing our understanding of how anthocyanins and other flavonoids impact health.

The authors have declared no conflict of interest.
